# Supplementary material for: A pipeline for the retrieval and extraction of domain-specific information with application to COVID-19 immune signatures
Source: BMC Bioinformatics. 2023 Jul 20;24:292. doi: 10.1186/s12859-023-05397-8 (PMC10357743; doi:10.1186/s12859-023-05397-8)
Supplement: Supplementary file 1 — Additional file 1. A copy of the generic pipeline (github.com/mcdougallab/pipeline) that has been adapted for COVID-19 immune signatures. [file 12859_2023_5397_MOESM1_ESM.zip › covid-pipeline-main/index.html]

The Human Immunology Project Consortium (HIPC) program was established in 2010, and renewed in 2015, by the NIAID Division of Allergy, Immunology, and Transplantation as part of the overall NIAID focus on human immunology. Through this program, well-characterized human cohorts are studied using a variety of modern analytic tools, including multiplex transcriptional, cytokine, and proteomic assays; multiparameter phenotyping of leukocyte subsets; assessment of leukocyte functional status; and multiple computational methods.

This website is an enhancement to leverage the HIPC infrastructure to capture COVID-19 related immune signatures. The rapid pace of COVID-19 related research has led to a large number of studies (both peer-reviewed and available through preprint servers) that carry out systems-level profiling to identify immune signatures.

These signatures will be curated from the literature and provide an important source of prior knowledge to guide the analysis of SARS-CoV-2 responses. They will also be used as a source of prior knowledge and validation of Immunophenotyping Assessment in a COVID-19 Cohort (IMPACC) findings.
