# Supplementary material for: A pipeline for the retrieval and extraction of domain-specific information with application to COVID-19 immune signatures
Source: BMC Bioinformatics. 2023 Jul 20;24:292. doi: 10.1186/s12859-023-05397-8 (PMC10357743; doi:10.1186/s12859-023-05397-8)
Supplement: Supplementary file 3 — Additional file 3. Supplementary tables of the tags and key words used in the pipeline, and the words frequency that differ between classes. [file 12859_2023_5397_MOESM3_ESM.pdf]

Supplementary Material

Tag	Terms
brain	brain, neurological, neuron, neurons
heart	heart, cardiac
kindey	kidney
liver	liver
lung	lung, pulmonary
artery	artery
skin	skin
bone	bone
upregulated	upregulated, up-regulated
downregulated	downregulated, down-regulated
rnaseq	rnaseq, rna-seq
immune response	immune response
t-cell	t cell, t cells, t-cell, t-cells
b-cell	b cell, b cells, b-cell, b-cells
virus	virus, viral
human	human, humans, patient, patients
antibody	antibody, antibodies
treatment	treatment, medicine
mouse	mouse, mice
macrophage	macrophage, macrophages
protein	protein, proteins
gene	gene, genes, genetic

Table 1: Abstracts containing any of the terms listed would be given the corresponding tag. Reviews can see these tags and search or filter abstracts with them.

immune signature	Immune signatures	Immune signature	IL-8
IL-12	B cells	B cell	T cells
T-cells	T-cell	T killer cells	T killer cell
NK cells	NK cell	macrophages	macrophage
IgA	CD4+	CD8+	cytokines
Cytokines	Cytokine	immunopathological	Immunopathological
immunophenotype	Immunophenotypes	Immunophenotype	immunology
immune reaction	Immune reaction	immune cells	immune cell
Immune cell	severe acute respiratory syndrome	Severe acute respiratory syndrome	COVID-19
blood samples	blood sample	inpatients	inpatient
non-human primate	nonhuman primates	nonhuman primate	macaques
Macaques	Macaque	humans	human
mass cytometry	proteomic	genomic	protein profile
flow cytometry	gene expression	single cell	review
vaccine	infections	infection	vaccination
Metatranscriptome	metatranscriptome	transcriptome	stimulation
in-vitro	in vivo	in-vivo	covid-19
SARS-CoV-2	SARS-CoV2	coronaviruses	coronavirus
influenza	Influenza	flu	Flu
mouse	mice	patients	patient
subject	single cell RNA-seq	single cell rna-seq	scRNA-seq
rna-seq	RNA-seq	elispot	EliSpot

Table 2: Terms identified by a reviewer that we highlight in the interface to facilitate classification.

words	relative frequency (%) with immune signatures	relative frequency (%) without immune signatures
**patient**	**6.45**	**0.98**
pandemic	0.13	0.54
**severe**	**1.92**	**0.16**
health	0.12	0.29
**cell**	**1.20**	**0.11**
**severity**	**0.86**	**0.05**
review	0.01	0.13
**blood**	**0.67**	**0.02**
**level**	**1.24**	**0.13**
testing	0.04	0.16

words	relative frequency (%) with type A signatures	relative frequency (%) with type B or C signatures
patient	3.10	14.21
clinical	0.46	3.35
hospital	0.05	2.07
covid	5.24	11.10
group	0.19	2.39
**gene**	**1.91**	**0.04**
admission	0.03	1.72
mortality	0.17	2.21
laboratory	0.02	1.25
risk	0.20	1.81

words	relative frequency (%) with type B signatures	relative frequency (%) with type A or C signatures
gene	0.03	1.63
covid	4.65	9.31
cell	1.22	3.26
patient	5.66	9.13
expression	0.09	0.95
lung	0.11	0.94
immune	0.45	1.58
pathway	0.05	0.66
injury	0.25	1.08
sars	0.80	2.02

words	relative frequency (%) with type C signatures	with type A or B signatures
cell	0.09	4.34
immune	0.05	1.96
response	0.09	1.68
sars	0.39	2.40
covid	4.78	9.02
severe	0.90	2.89
infection	0.35	1.69
cytokine	0.03	0.85
pathway	0.01	0.70
disease	1.24	3.05

Table 3: **Differences in relative frequencies between papers predicted to have COVID-19 immune signatures and the signature type.** The ten words with largest differences in relative word frequencies are shown. Bold text indicates the word had a higher relative frequent in the set, light text that it was less frequent. Relative frequencies are given as the occurrences of the word as a percent of the corpus.
